# How do neighborhood environments impact adolescent health: a comprehensive study from subjective and objective perspectives using machine learning method

**DOI:** 10.3389/fpubh.2024.1507711

**Published:** 2025-01-03

**Authors:** Jie Sheng, Zhenhai Xiang, Pengfei Ban

**Affiliations:** School of Architecture and Urban Planning, Kunming University of Science and Technology, Kunming, China

**Keywords:** neighborhood environment, random forest, adolescent, physical health, mental health, non-linear relationship

## Abstract

Existing studies have established a linear relationship between urban environments and adolescent health, but the combined impacts of subjective and objective environments on multi-dimensional health status (including physical and mental health) have not been fully explored. Furthermore, while some studies have examined the non-linear relationship between urban environments and adult health, research specifically focusing on adolescents is sparse. Using Kunming, China, as a case study, we employ Random Forest model to examine the non-linear relationship between subjective/objective neighborhood environments and adolescent physical/mental health. The results indicate that the objective environment plays a more significant role in influencing physical and mental health in adolescents. There are generally non-linear correlations and threshold effects between neighborhood environment variables and adolescents’ health status. Specifically, the effects of distance to subway station, ratio of traffic safety facilities, and greening view index on adolescent physical and mental health differ. Additionally, subjective environments characterized by community management, community image, and community capital tend to positively influence adolescents’ health status. This study provides valuable insights for the planning of healthy communities, environmental interventions, and health promotion in specific dimensions among adolescents.

## 1 Introduction

Physical and mental health issues have become significant obstacles for adolescents during their physical and emotional development, as well as their cognitive ability formation stages. Studies indicate that between 2015 and 2019, 11.1% of Chinese children and adolescents aged 6 to 17 were overweight, with 7.9% classified as obese (1). A national survey conducted in 2016 revealed that the rate of students in China meeting the standard for physical health was only 23.8% (2). Additionally, evidence suggests that the prevalence of anxiety and depression among adolescents is approximately 4.7 and 3.0% (3), respectively, with the overall prevalence of depression exceeding the global average (4). As the world’s second most populous country after India, China has more than 158 million adolescents (5), highlighting the significance of these health issues.

The effective intervention in neighborhood environments to promote positive health status for adolescents has become a significant topic in fields such as urban planning, geography, and transportation. Existing research typically measures neighborhood environments from two perspectives: the objective environment and subjective environment. On one hand, the widespread use of geographic information systems and urban spatial data has facilitated extensive studies on the relationship between objective physical characteristics and health, such as blue and green spaces, road design, land use mix, and accessibility of services and transportation facilities (6–8). However, the objective environmental measurements are mostly overall depictions of the surrounding community environment and are difficult to reflect the perceptual circumstances under individual differences in needs. Therefore, on the other hand, through interviews and questionnaire surveys, some studies have explored the relationship between individual environmental perception and health, such as assessments and perceptions of environment and facilities, neighborhood order and community support (9–11). Nevertheless, while both objective and subjective environmental factors have shown significant effects on adolescent health, only a few studies have explored the combined impact on health.

Most existing studies on the built environment and residents’ health are based on linear assumptions. Nevertheless, the influences of built environment elements may fluctuate depending on their scope. As a result, concentrating on a sole relationship and disregarding potential non-linear effects may bring about disparities in results (12). In recent years, with the application of machine learning in the domains of urban planning, geography, and transportation, numerous studies have explored the complex non-linear relationship between the built environment and health. For instance, some evidence has revealed that environmental elements such as greenness and population density have complex effects on residents’ health, including inverted U-shaped and N-shaped relationships (13–16). Nevertheless, the limited research has merely investigated the relationship between the built environment and adult health, while there has been virtually no exploration concerning the relationship between the built environment and the health of adolescents who are in the stage of physical and mental development.

In summary, existing research has predominantly examined the relationship between neighborhood environments and adolescent health from a singular perspective, focusing either on the objective or subjective environment. However, there is a notable lack of studies assessing the relative importance and differing impacts of both environments on various health dimensions. Furthermore, research addressing the nonlinear associations between neighborhood environments and health outcomes is limited, with most studies concentrating on adults and offering little insight into the experiences of adolescents.

Therefore, this study uses data from the “Questionnaire Survey on Primary and Secondary School Students’ Commuting and Healthy Growth” in Kunming, China, along with multi-source urban spatial data. By employing Random Forest model, it aims to explore the non-linear associations between subjective/objective environments and adolescent physical/mental health. This study attempted to address two questions: (1) What are the contributions of environmental characteristics to adolescent health, and are there differences between subjective and objective environments? (2) Do subjective and objective environments demonstrate a non-linear relationship and threshold effects with adolescent physical and mental health?

## 2 Literature review

### 2.1 Objective/subjective environment and adolescent health

The concept of “Healthy Cities” was formally introduced by the World Health Organization (WHO) in 1984, emerging from research on the impact of public health systems on residents’ well-being. One of its definitions is that a healthy city should create and improve physical and social environments, and enhance social support through expanded community resources (17). With the advancement of the “Healthy Cities” movement, theoretical discussions between environment and health have increased. Among these, socioecological theory, which provides a framework for sustaining the “individual-environment” system, emphasizes the interactive relationship between individuals and environment (18). It posits that health is influenced by multiple layers, including personal and environmental factors, where environmental factors include the built, natural, and social environment (18, 19). Based on the theoretical explanation of “environment-health,” numerous scholars have explored the mechanisms through which multidimensional environments impact health. Among these, the neighborhood environment, where urban residents (particularly adolescents) live for extended periods, has been widely recognized as having a significant impact on health. Recent studies on neighborhood environments and adolescent health commonly measure neighborhood conditions in two dimensions, objective and subjective environments, due to differences in research content and measurement approaches (such as urban spatial big data or questionnaire survey data).

On one hand, the objective environment reflects the physical spatial structure of the community and the opportunities for accessing service facilities, and it can be categorized into natural and built environment based on specific indicators and research focus. The natural environment includes the density, quality, and exposure levels of blue and green spaces (13, 20–22). The built environment includes the road design (e.g., street connectivity, road and intersection density) (7, 8, 13, 23), density and accessibility of service facilities (e.g., parks, restaurants, and sports and recreational facilities) (6, 23, 24), land use mix (7, 25, 26), density and accessibility of transportation (e.g., bus stop and subway station) (7, 8, 13, 25). Regarding the natural environment, appropriate exposure to nature and well-designed landscapes can positively influence adolescents’ physical and mental health. For instance, the coverage and proximity to parks, forests, or green spaces have been shown to improve sleep efficiency, reduce body mass index (BMI) (6, 20, 27), and improve mental well-being (including reductions in depression, stress, anxiety, and behavioral health issues) (28, 29). Regarding the built environment, it influences adolescent health by shaping the overall quality of the community (e.g., residential quality, land use mix, and facility distribution). For instance, mixed land-use patterns (7, 25), appropriate intersection density (25), and the proximity of parks and sports facilities (7) promote physical activity, support social interactions, and reduce sedentary behavior (24), thus positively influencing adolescents’ physical health and emotional well-being. Conversely, significant environmental pollution, including air and noise pollution, can adversely affect physical health and lead to negative emotions (9, 20, 30). For instance, excessive concentrations of pollutants like NO_2_ and PM_2.5_, negatively affect adolescent sleep health (20), and are associated with adolescent psychiatric experiences (31). Although the objective environment effectively represents the physical spaces that adolescents inhabit, it does not fully capture the comprehensive subjective assessments of environmental factors that influence health status or behaviors (32, 33).

On the other hand, the subjective environment reflects adolescents’ perceptions and evaluations of the physical and social contexts, which indicates that individual differences may result in varied interpretations of similar neighborhood environments. This includes assessments and perceptions of the natural environment (10, 34), built environment (e.g., walkability, cognitive aspects of buildings, and service facilities) (10, 35), neighborhood order (e.g., crime and safety) (11, 36, 37), community support and interaction (e.g., cohesion, trust, and social capital) (6, 9, 38, 39). Among them, perceptions of the physical environment can indirectly influence adolescent health by promoting or hindering physical activity levels. For instance, perceived favorable landscape conditions (10) and proximity to recreational facilities (35) provide opportunities for positive interactions with the environment, thereby fostering healthy emotional responses and habits. In contrast, perceptions of the social environment significantly impact adolescents’ social behaviors and interactions. For example, perceived community safety is positively associated with reductions in adolescents’ body mass index (BMI) and well-being (6, 36). Conversely, negative perceptions of neighborhood trust, social cohesion, and social capital are associated with a higher likelihood of adolescents reporting internalizing and externalizing problem behaviors (40, 41).

Previous studies highlight that both objective and subjective environments are critical factors influencing adolescent health. Recognizing the limitations of assessing environmental influences on adolescent health through a single-dimensional approach, a few studies have started to examine the relationship between the environment and health by considering both subjective and objective environmental factors (6, 9, 42). However, variations in indicator selection and research focus have led these studies to primarily explore the effects of significant environmental variables. Recently, studies on neighborhood environment and residents’ health have increasingly focused on the joint analysis of objective and subjective characteristics (10, 33, 43, 44). Due to differences in measurement approaches and representational meanings, the relative influence of objective and subjective environments on health may vary. For instance, certain study indicates that the objective environment may be more significant (45), as it reflects the actual physical space of the community, thereby confirming the real meaning of the perceived environment and helping to guide planning practices (33). Other studies, however, indicate that the subjective environment is more significant (15, 43, 46), as perceptions of the environment reflect individual cognitive processes and have a particularly significant impact on health (47). Although the relative importance may vary by research aim and context, both objective and subjective environments contribute to reflecting the significant influence of the built environment on health through environmental perception and the promotion of health behaviors.

In summary, using indicators from both subjective and objective environments provides a comprehensive understanding of the neighborhood environments that adolescents experience. However, existing research often examines the relationship between neighborhood environments and adolescent health from a singular perspective (subjective or objective) and single dimension (such as two-dimensional spatial environment). Few studies have investigated the interplay between subjective and objective environments.

### 2.2 Non-linear association between environment and health

Existing research has mainly investigated the impact of neighborhood environment on adolescent health using linear assumptions, often focusing on single effects. However, variations in indicators and methodologies frequently lead to estimation bias (48, 87). For instance, natural environmental contact has been shown to have a positive effect on health in most studies, and air pollution has a negative effect. But these two environmental conditions have not been able to predict mental health outcomes in some studies (42). Likewise, while proximity to transit stations and road density are generally positively associated with adolescent health, some studies have found no significant correlations with physical health outcomes (7). Additionally, the effects of built environment elements may differ by spatial scale, and focusing only on linear relationships could overlook potential nonlinear effects, leading to inconsistencies in findings (12). For instance, land use mix is generally positively correlated with adolescent physical and mental health (7, 25), but another study indicates that its impact on adolescent BMI is not significant, and may exhibit a possible nonlinear relationship (26). In the only two studies we found that explore the non-linear relationship between neighborhood environment and adolescent health, the non-linear relationships and threshold effects of certain environmental variables have been confirmed. For instance, the distance to transit stations shows a locally significant positive effect on adolescent physical health within specific intervals (7, 13), while green spaces (e.g., park coverage and NDVI) are associated with reduced adolescent obesity rates within certain thresholds (13).

Most existing studies on the built environment and residents’ health are based on linear assumptions. Recently, there has been growing interest in the non-linear associations between neighborhood environments and various health-related factors, including health status (15, 49, 50), obesity (13, 51, 52), and positive health behaviors (16, 53, 54). Some studies have identified potential threshold effects for specific built environment variables, indicating that significant effects only emerge within certain ranges. For instance, optimal levels of built environment features, such as proximity to sports facilities and bus stops (15), road density (54), and land use mix (53), can effectively enhance residents’ physical and mental health while promoting active travel. Moreover, evidence suggests that the relationships between certain built environment factors and health outcomes can vary across different ranges of influence. For example, green exposure has been widely shown to have complex mechanisms affecting health. Adequate levels of green exposure offer more opportunities for interaction with nature and improved landscape conditions, fostering positive health outcomes and related behaviors. However, negative impacts may arise beyond certain thresholds (12, 13, 15). Similar effects have been observed with road connectivity (15, 55), bus stop density (14, 15), and population density (13, 14).

## 3 Materials and methods

**Figure 1:**
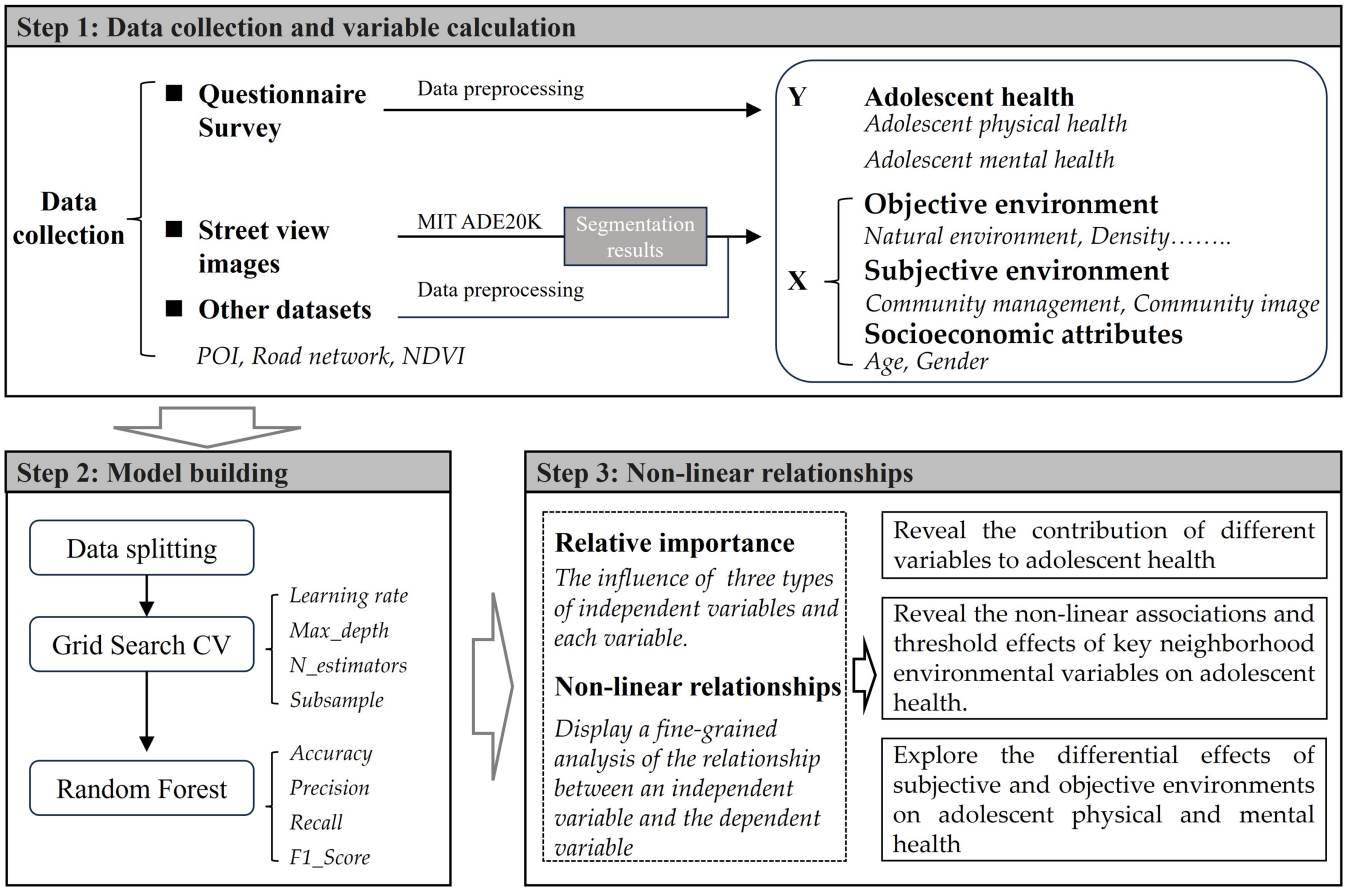
Framework.

The three-step workflow in this study is shown in Figure 1. Firstly, this study uses data from the “Questionnaire Survey on Primary and Secondary School Students’ Commuting and Healthy Growth” in Kunming, China, along with street view image and multi-source urban spatial data. Secondly, by employing Random Forest model, it evaluates the relative importance of subjective environments, objective environments, and socioeconomic attributes on different dimensions of adolescent health. Additionally, it reveals the non-linear associations and threshold effects of key neighborhood environmental variables on adolescent health.

### 3.1 Study area and data

**Figure 2:**
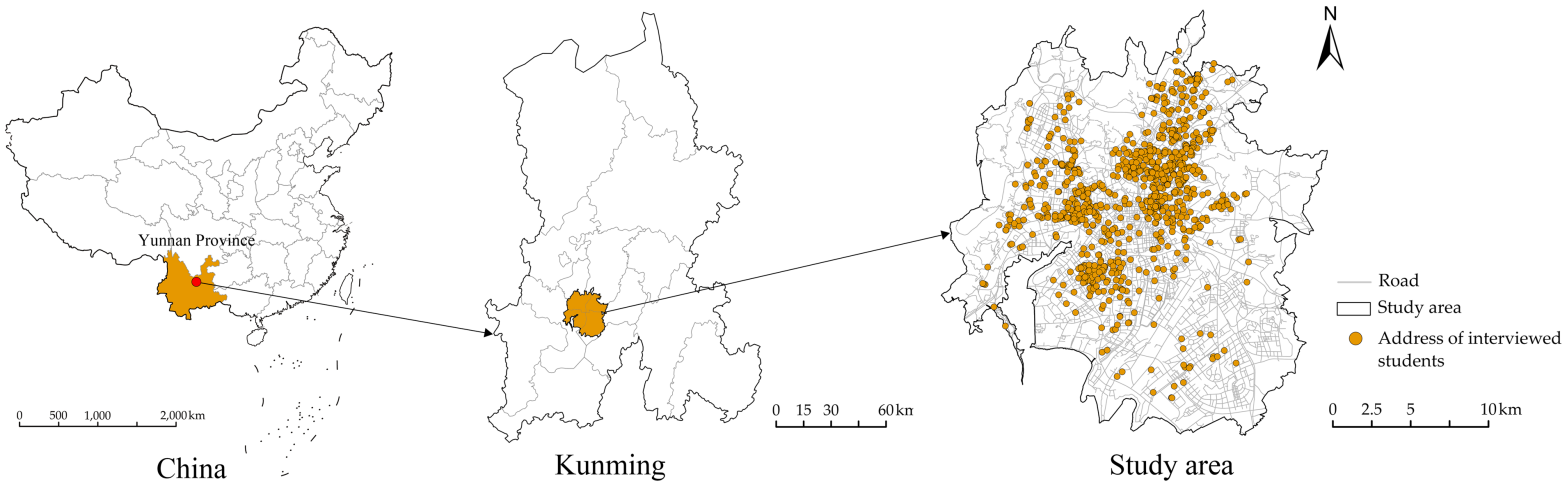
Study area.

Kunming, the capital of Yunnan Province, serves as a key gateway to South and Southeast Asia and is one of the core cities in Southwest China. As of 2023, Kunming has a resident population of 8.68 million (56), and covers an area of 21,012.54 km^2^ (57). The study area is defined by the spatial scope outlined in the “Urban Master Plan of Kunming (2011–2020).” It includes the area to the east of the Third Ring Road and enclosed by the Ring Expressway. This region encompasses the core built-up areas of Kunming’s four main districts (Wuhua, Panlong, Xishan, and Guandu) and serves as a significant hub for the city’s public service facilities (Figure 2).

The data for this study were obtained from the “Online Questionnaire Survey on Primary and Secondary School Students’ Commuting and Healthy Growth,” conducted in Kunming, China in January 2024. The survey covered five major districts in Kunming: Wuhua, Panlong, Xishan, Guandu, and Chenggong. First, we randomly selected 31 sample schools from a total of 402 primary and middle schools in five districts using a stratified sampling strategy (these schools are within the compulsory education stage). Subsequently, we contacted the Kunming Education and Sports Bureau to obtain permission for the online survey distribution and coordinated with the teachers in charge of each school. We randomly selected approximately 5% of students from grades 1–6 in primary schools or grades 1–3 in middle schools in the sample schools, and distributed a total of 1831 questionnaire QR codes. The online questionnaires were completed jointly by students and their parents. Given that younger students may not fully understand the questions, we specifically noted that sections to be filled out by students should be completed with parental assistance. The legal guardians of all participants signed informed consent forms after being briefed on the purpose of the survey, and their information is strictly protected in accordance with relevant regulations. The survey collected information on various aspects, including students’ personal and family backgrounds, health status, and subjective environmental perceptions. After excluding questionnaires with incomplete information (such as home and school addresses), anomalies, and those with low reliability, we obtained 1,583 valid questionnaires, yielding an effective rate of 86.45%. These samples were distributed across 606 residential communities in the five districts. Given the limitations and representativeness of the urban built environment data, we further refined the sample to 1,388 valid responses within the study area. Research indicates that children aged 7 to 12 develop more logical and organized ways of thinking compared to children under the age of 7 (58). Therefore, to more accurately capture adolescents’ self-perception of the environment and health conditions, we excluded students under 7 years of age, yielding a final sample of 1,355 for subsequent analysis.

In addition to the survey data, we collected the following multi-source urban spatial data: (1) Road network data was obtained from OpenStreetMap.^1^ The raw road network data were processed using ArcMap 10.8.1, including clipping, cleaning, and topological adjustments, with interruptions at intersections. (2) Points of Interest (POI) data was acquired from the Gaode Map.^2^ (3) Street view image was collected from the Baidu Map.^3^ A Python program was used to sample street view panoramic images at 100-meter intervals from the road network, resulting in 54,431 images. These images were analyzed using a PSP Net model pre-trained on the MIT ADE20K dataset to identify and calculate the area proportions of various visible spatial elements, such as sky, roads, and vegetation. (4) Normalized difference vegetation index (NDVI) was obtained from the National Ecological Science Data Center.^4^

### 3.2 Variables

#### 3.2.1 Dependent variables

The dependent variables include participants’ self-reported status for physical and mental health. In terms of the physical health, students were asked, “How would you rate your physical health?,” with responses recorded on a Likert scale from 1 (very poor) to 5 (very good). In terms of the mental health, we used the “Chinese Adolescents’ Emotional and Behavioral Problems Simplified Scale,” which has demonstrated high reliability and validity in a survey involving 4,727 students in Hunan Province, China (59). We assessed mental health across three dimensions: anxiety, depression, and social problems. Each dimension comprises 10 to 13 questions, with response options and scoring as follows: Never/Rarely (1), Sometimes (2), Often (3), and Most of the time (4). Notably, since most response options are negative indicators, we converted all responses to positive values for the calculation of mental health scores, thereby measuring positive levels of mental health.

In this study, we assessed the relative health status of the surveyed students. First, participants who rated their physical health as “good” or “very good” were classified as “healthy,” while those with remaining ratings were classified as “unhealthy” (15). Second, for mental health, we calculated the average scores for each dimension across all questions. Participants with average scores below 3 were classified as “unhealthy,” while those with scores above 3 were classified as “healthy.”

#### 3.2.2 Independent variables

**Figure 3:**
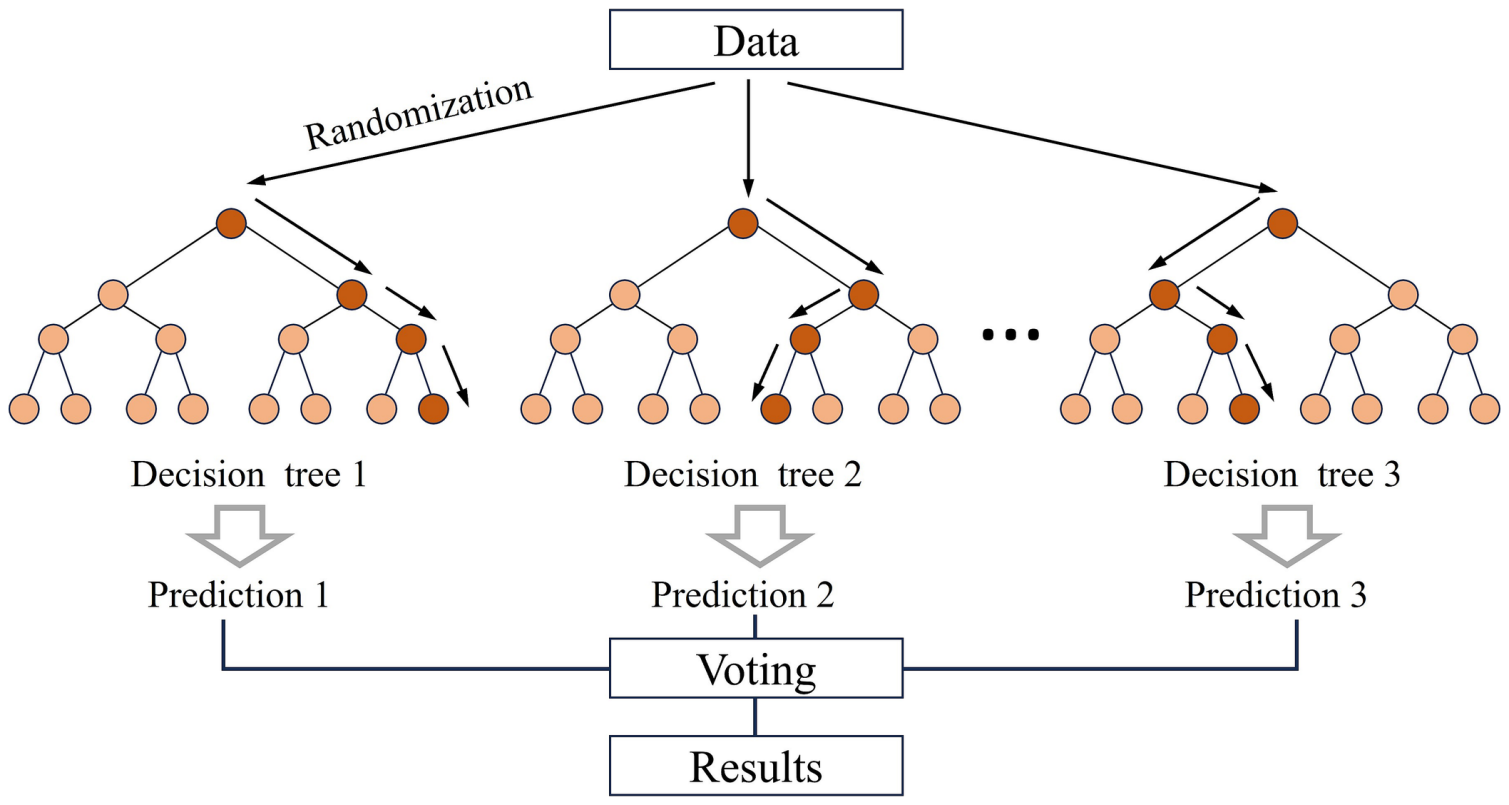
The process of random forest algorithm [adapted from Xu et al. (15)].

Based on the multidimensional definitions of environmental factors influencing health in Healthy Cities theory and socioecological theory (including the natural, built, and social environments), along with extensive discussions on objective and subjective environments in neighborhood studies on adolescent health, we developed an analytical framework for examining the impact of neighborhood environments on health (Figure 3). Regarding the neighborhood environment, we referenced the classification of residential environment attributes into subjective and objective components in the neighborhood satisfaction model (60), and reviewed evaluation variables related to both objective environments and subjective perceptions in existing research. A total of 26 indicators were selected, covering aspects of the objective environment, subjective environment, and socioeconomic attributes (Table 1). The following sections will provide a detailed introduction to the variables for both objective and subjective environments.

**Table 1:** Description of variables.

Variables		Description	Mean (standard deviation) or proportion
Objective environment	Natural environment		
	NDVI	The mean of NDVI within 0.8 km buffer	0.40 (0.05)
	Density		
	Intersection density (number/km^2^)	Number of road intersections / buffer area	19.47 (6.81)
	Diversity		
	Land use mix	$\mathrm{Land}\;\mathrm{use}\;{\mathrm{mix}}_{i}=-\frac{1}{\ln A}\overset{A}{\underset{j=1}{\sum }}p_{ij}\ln p_{ij}$ Where $p_{ij}$ refers to the proportion of the j -th type of POI within unit i relative to the total number of POIs in that unit. A refers to the number of POI types in the unit.	0.74 (0.05)
	Street design		
	Sky view index (%)	The mean of sky pixel ratio within 0.8 km buffer	48.85 (4.02)
	Greening view index (%)	The mean of green plants pixel ratio within 0.8 km buffer	12.14 (2.59)
	Relative pedestrian width	The mean ratio of pedestrian pathways to roads within 0.8 km buffer	0.37 (0.88)
	Ratio of traffic safety facilities (%)	The mean of safety facilities ratio within 0.8 km buffer	0.81 (0.22)
	Distance to transit		
	Distance to bus stop (km)	Distance to the nearest bus stop	0.25 (0.18)
	Distance to subway station (km)	Distance to the nearest subway station	1.42 (0.81)
	Destination accessibility		
	Distance to sports facility (km)	Distance to the nearest sports facility	0.36 (0.35)
	Distance to park or square (km)	Distance to the nearest park or square	0.69 (0.51)
Subjective environment	Community management		
	Perceived sanitary condition	1 (Very bad) to 5 (Very good)	3.74 (0.87)
	Perceived property management quality	1 (Very bad) to 5 (Very good)	3.50 (0.98)
	Community safety	1 (Very bad) to 5 (Very good)	4.01 (0.83)
	Community image		
	Subjective noise quality	1 (Very noise) to 5 (Very quiet)	3.50 (1.00)
	Subjective air quality	1 (Very bad) to 5 (Very good)	3.72 (0.88)
	Perceived landscape environment	1 (Very bad) to 5 (Very good)	3.51 (0.90)
	Community capital		
	Sense of community belonging	1 (Very weak) to 5 (Very strong)	3.49 (0.89)
	Neighborhood familiarity	1 (Incognizant) ~ 5 (Knows each other)	2.29 (0.97)
	Frequency of community events	1 (None/very few) ~ 5 (Frequently)	2.59 (1.10)
Socioeconomic attributes	Age	The age of surveyed students	11.02 (2.18)
	Gender	Male = 0, Female = 1	0 = 52.77%, 1 = 47.23%
	Household registration	Non-local household registration =0, Local household registration = 1	0 = 36.97%, 1 = 63.03%
	Household income level (RMB)	Yearly household income: <50,000 = 1, 50,000–150,000 = 2, 150,000-250,000 = 3, > 250,000 = 4	2.29(0.93)
	Housing area(m^2^)	Housing area of surveyed students	101.11 (53.01)
	Car ownership status	Without private vehicle = 0, With private vehicle = 1	0 = 16.01%, 1 = 83.99%

##### 3.2.2.1 Objective environment

Previous research on objective environment and adolescent health consistently highlights the significant health impacts of factors such as greening levels, road design elements, and built environment attributes. These factors influence adolescent health both directly and indirectly by shaping landscape quality, residential conditions, service accessibility, and transportation connectivity, ultimately impacting health behaviors and social interactions. Recent advancements in street view image semantic segmentation via machine learning have enhanced understanding of the built environment by measuring three-dimensional street visual elements, which have increasingly been applied in studies on adolescent health behaviors.

Based on previous studies and the widely used “5Ds” model in built environment and health research, we measured the objective environment using 11 variables across five dimensions. We created a circular buffer with 800-meter radius, commonly referred to as a 15-min pedestrian-scale neighborhood, within which residents can generally access essential service facilities. The natural environment is measured by NDVI (13, 15), reflecting adolescents’ access to natural surroundings. Density is measured by intersection density (8, 25), reflecting the street network layout within the community. Diversity is measured by land use mix, reflecting the variety of functional facilities within the community (7, 25, 26). Street design is evaluated through the sky view index, greening view index, relative pedestrian width, and ratio of traffic safety facilities (61, 62), reflecting the three-dimensional visual conditions encountered by adolescents on streets. These indicators were determined by averaging the pixel ratio from street view images at each sampling point. Accessibility to transportation and facilities is measured by the distance to nearest bus stop, subway station, park or square, and sports facilities, reflecting adolescents’ access to transportation, natural or open spaces, and recreational spaces in the neighborhood (7, 13, 20). These indicators were determined using the tool of “Find Closest Facilities” in ArcMap 10.8.1.

##### 3.2.2.2 Subjective environment

Similarly, previous studies have demonstrated that the perceptions of natural, built, and social environments significantly impact adolescent health by fostering positive lifestyles and attitudes. Based on these studies, we measured the subjective environment using 9 variables across three dimensions. Community management is measured by perceived sanitary condition and property management quality, and community safety (11, 36, 37, 63), reflecting perceptions of community management quality and safety. Community image is measured by subjective air quality and noise quality, and perceived landscape environment (10, 34), reflecting perceptions of the neighborhood’s physical environment. Community capital is measured by sense of community belonging, neighborhood familiarity, and frequency of community events (6, 9, 38, 39), reflecting social support and interpersonal interactions within the community. All subjective environmental indicators were evaluated using a five-point Likert scale.

### 3.3 Method

**Figure 4:**
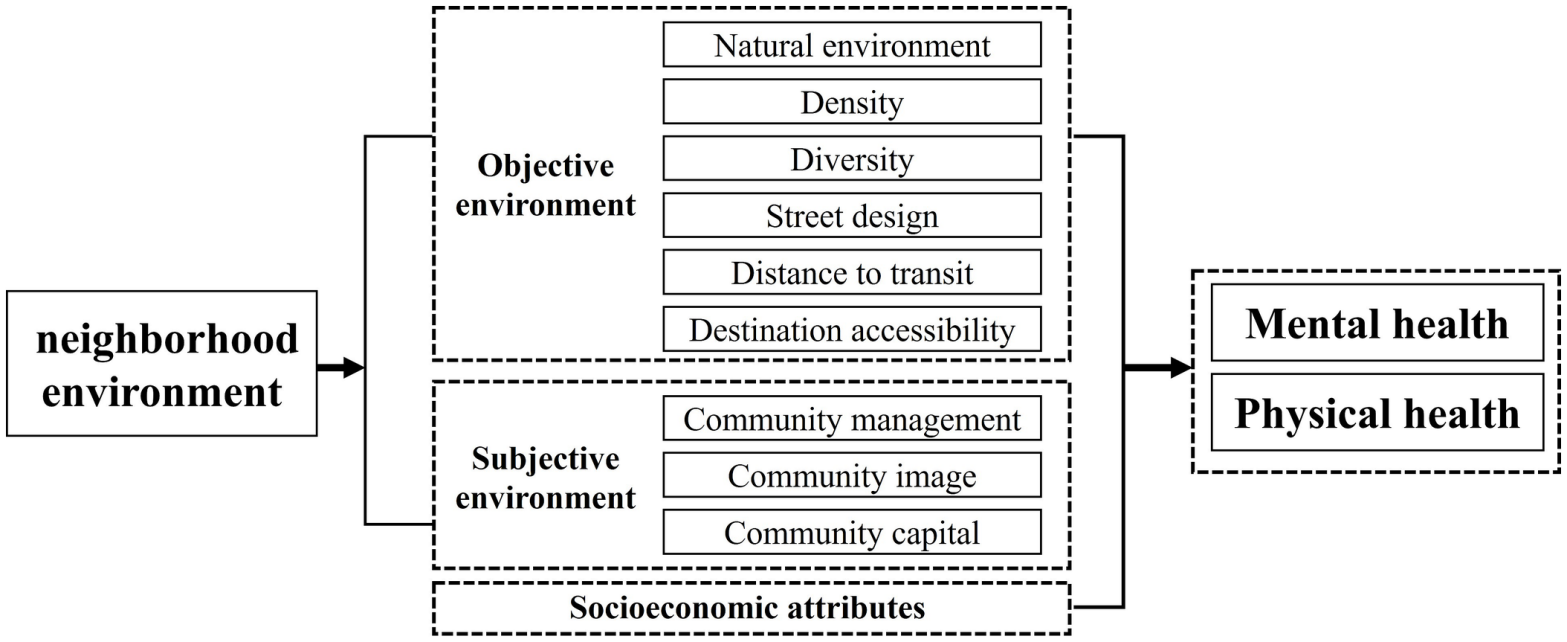
The framework for neighborhood environments on health.

The Random Forest (RF) model, introduced by Leo Breiman in 2001, is a decision tree-based machine learning algorithm, particularly effective at handling large datasets while minimizing the risk of overfitting (64). In classification tasks, as illustrated in Figure 4, the core principle involves repeatedly drawing M sample sets from the original dataset using the bootstrap resampling method, generating M decision trees that collectively form a random forest. At each tree node, a random subset of n features is selected from the total of N features, and the feature that minimizes the Gini index is chosen to split the node. Each tree is fully grown, and the ensemble of M trees provides the final classification result, which is determined by majority voting across all trees.

In this study, we utilized the “Random Forest “package in Python 3.6, Jupyter Notebook 5.7.10. The model construction and validation process involves two main steps. First, using the ‘train_test_split’ module randomly allocates 70% of the analysis samples to the training set for model building, while the remaining 30% are reserved as the test set to assess the model’s performance. Second, to optimize the model and avoid issues of underfitting and overfitting, based on previous study (65), three key hyperparameters are fine-tuned. The search ranges for these hyperparameters are as follows: max_depth ranging from 3 to 7 (step size of 1); n_estimators ranging from 100 to 500 (step size of 100); and max_features ranging from 1 to 5 (step size of 1). Grid SearchCV from Scikit-learn is used for grid search and five-fold cross-validation to evaluate 125 possible hyperparameter combinations and determine the optimal set that delivers the best model performance: max_depth is set to 3, n_estimators is set to 100, max_features is set to 1.

## 4 Results

### 4.1 Model performance

To assess the suitability of the Random Forest model for this study, we trained and tested three commonly used machine learning models: RF, XGBoost, and LightGBM. The performance of these models is evaluated using commonly used metrics in supervised machine learning: Accuracy, Precision, Recall, and F1 scores. The value closer to 1 indicates better model performance (13, 15). As shown in Table 2, RF outperformed the other models in accuracy, recall, and F1 score. Although RF did not achieve the highest precision value among the models, it was very close to the maximum value. Overall, the Random Forest model demonstrated high accuracy, adaptability, and excellent predictive performance, making it effective for uncovering the complex nonlinear relationships between neighborhood environment and adolescent health. To further assess the robustness of the selected research units and study results, we constructed an 800-meter network buffer to measure the objective environment. The results indicate that the Random Forest model also exhibits strong robustness. Furthermore, the relative importance rankings of both subjective and objective environmental factors remained largely consistent. Specifically, with the exception of a few variables, the rankings of the top 12 factors remained stable across each control group.

**Table 2:** Model performance metrics.

Model		Accuracy	Precision	Recall	F1 scores
Physical_health	Radom forest	**0.8771**	**0.8837**	**0.9917**	**0.9346**
	XGBoost	0.8570	0.8832	0.9660	0.9231
	LightGBM	0.8649	0.8822	0.9778	0.9275
Mental_health	Radom forest	**0.8157**	0.8210	**0.9910**	**0.8985**
	XGBoost	0.8030	0.8244	0.9670	0.8901
	LightGBM	0.7985	0.8203	0.9672	0.8877

### 4.2 Relative importance of predictors

Table 3 displays a comparison of the relative importance and rankings of all variables. Among the three types of independent variables, the objective environment has the greatest significance for both physical and mental health, with relative importance percentages of 51.03 and 53.30%, respectively. The subjective environment ranks next in importance, with its significance notably exceeding that of socioeconomic attributes. When comparing the relative importance rankings and percentages of various indicators, the following observations are made: (1) The greening view index, relative walking width, and ratio of traffic safety facilities in representing the three-dimensional environment of streets are comparable. (2) In contrast, the NDVI is more strongly associated with physical health, whereas intersection density, distance to bus stop, and community safety are more strongly associated with mental health.

**Table 3:** Relative importance of variables.

Variables	Physical health		Mental health	
	Relative importance (%)	Rank	Relative importance (%)	Rank
**Objective environment**	**51.03%**		**53.30%**	
NDVI	4.02%	11	2.39%	20
Intersection density	3.12%	17	5.75%	5
Land use mix	4.76%	7	5.72%	7
Sky view index	4.86%	6	4.03%	13
Greening view index	5.37%	4	5.73%	6
Relative pedestrian width	5.17%	5	5.29%	8
Ratio of traffic safety facilities	4.36%	8	4.40%	9
Distance to bus stop	3.66%	14	6.98%	1
Distance to subway station	7.84%	2	6.55%	3
Distance to sports facility	3.64%	15	2.32%	21
Distance to park or square	4.22%	10	4.14%	11
**Subjective environment**	**33.61%**		**32.92%**	
Perceived sanitary condition	2.02%	23	2.81%	19
Perceived property management quality	3.73%	13	3.09%	17
Community safety	1.97%	24	4.05%	12
Subjective noise quality	4.28%	9	4.16%	10
Subjective air quality	3.98%	12	3.49%	15
Perceived landscape environment	2.86%	19	2.18%	22
Sense of community belonging	9.08%	1	6.07%	4
Neighborhood familiarity	2.18%	22	3.68%	14
Frequency of community events	3.52%	16	3.38%	16
**Socioeconomic attributes**	**15.36%**		**13.78%**	
Age	0.72%	25	0.79%	25
Gender	3.10%	18	1.78%	23
Household registration	2.30%	21	0.56%	26
Household income level	2.80%	20	2.97%	18
Housing area	5.92%	3	6.66%	2
Car ownership status	0.53%	26	1.03%	24

In terms of physical health, the most significant variables in the neighborhood environment are sense of community belonging, distance to subway station and greening view index. The relative walking width, sky view index and land use mix are also closely associated with physical health. However, the relative importance of community safety and perceived sanitary condition is comparatively lower.

In terms of mental health, the most significant variables are distance to bus stop and subway station, sense of community belonging. Next in importance are intersection density, greening view index, and land use mix. Conversely, the relative importance of perceived landscape environment and distance to sports facility is comparatively lower.

Additionally, among socioeconomic attributes, housing area plays a significant role in influencing the physical health of adolescents, with its relative importance ranked 3rd for physical health and 2nd for mental health. Other personal socioeconomic attributes, such as gender and household registration, do not exhibit a significant impact on adolescent health.

### 4.3 Non-linear relationship

We visualize the non-linear relationships and threshold effects between various variables and adolescent physical and mental health using partial dependence plots. Additionally, we apply a fitting curve to smooth the effects of objective environmental factors and some socioeconomic attributes on health outcomes (66). Based on the relative importance ranking of factors influencing adolescent health, we individually analyze the top 12 most significant neighborhood environment variables.

#### 4.3.1 Adolescent physical health

**Figure 5:**
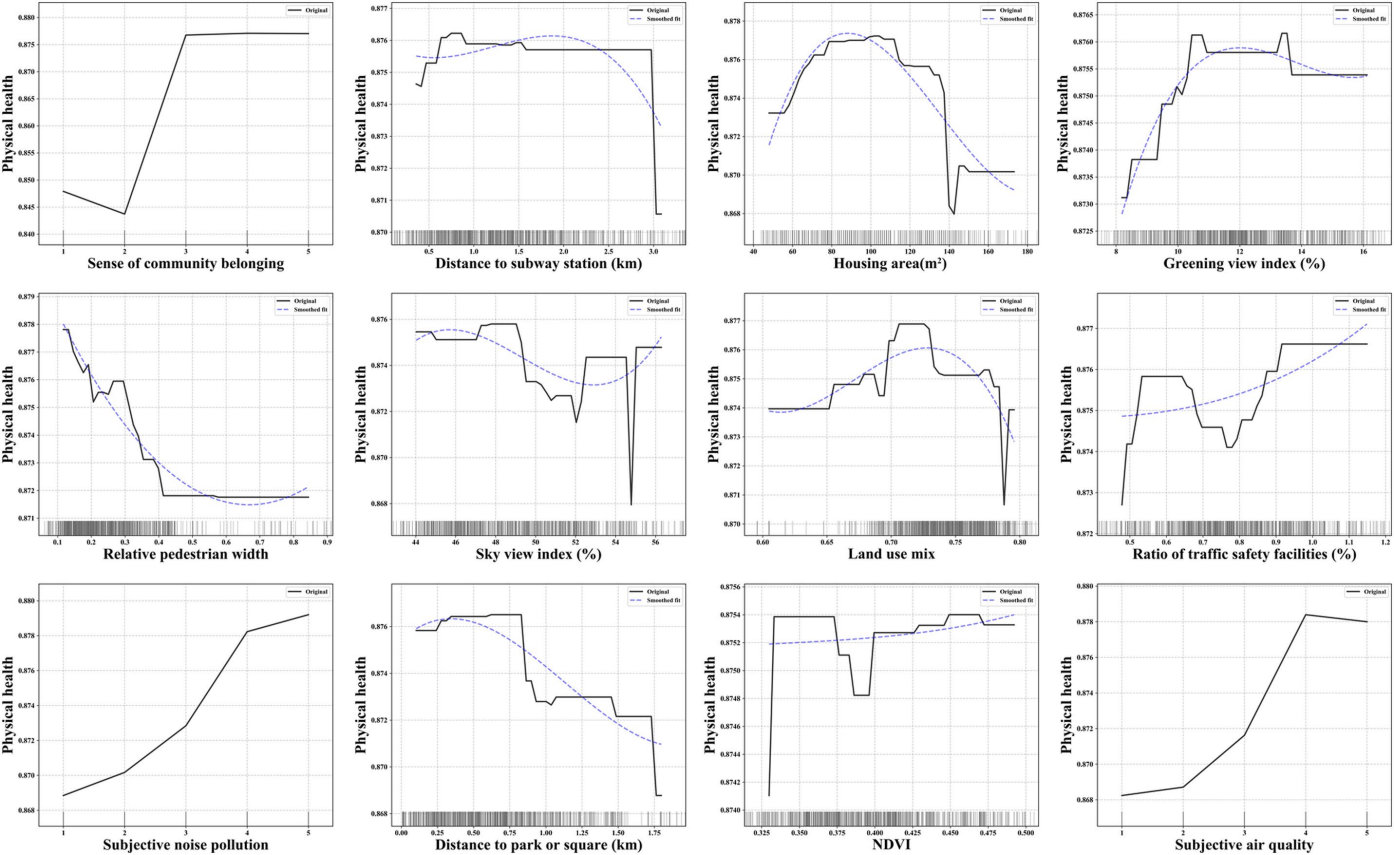
The non-linear association between key variables and adolescent physical health.

Figure 5 displays the partial dependence plots for the top 12 variables influencing adolescent physical health. In terms of the objective environment, the greening view index, ratio of traffic safety facilities and NDVI are positively associated with the probability of positive physical health status, with evident threshold effects. Specifically, the greening view index shows no significant effect once it exceeds approximately 14%, while ratio of traffic safety facilities and NDVI exhibit local low values around 0.8 and 0.38, respectively. Conversely, the relative walking width, distance to subway station and park or square are negatively associated with the probability of positive physical health status. Specifically, the relative walking width shows no significant effect once it exceeds approximately 0.4. Additionally, the sky view index and land use mix exhibit differentiated relationships with the probability of positive physical health status. Specifically, the sky view index leads to a “decrease–increase” trend in the probability, with a threshold at approximately 52%. The land use mix leads to an “increase–decrease” trend in the probability, with a threshold at approximately 0.71.

In terms of the subjective environment, the sense of community belonging, subjective noise quality and air quality are positively associated with the probability of positive physical health status. Specifically, the probability is maximized when sense of community belonging is rated as “normal,” subjective noise quality is rated as “very quiet,” and subjective air quality is rated as “good.”

#### 4.3.2 Adolescent mental health

**Figure 6:**
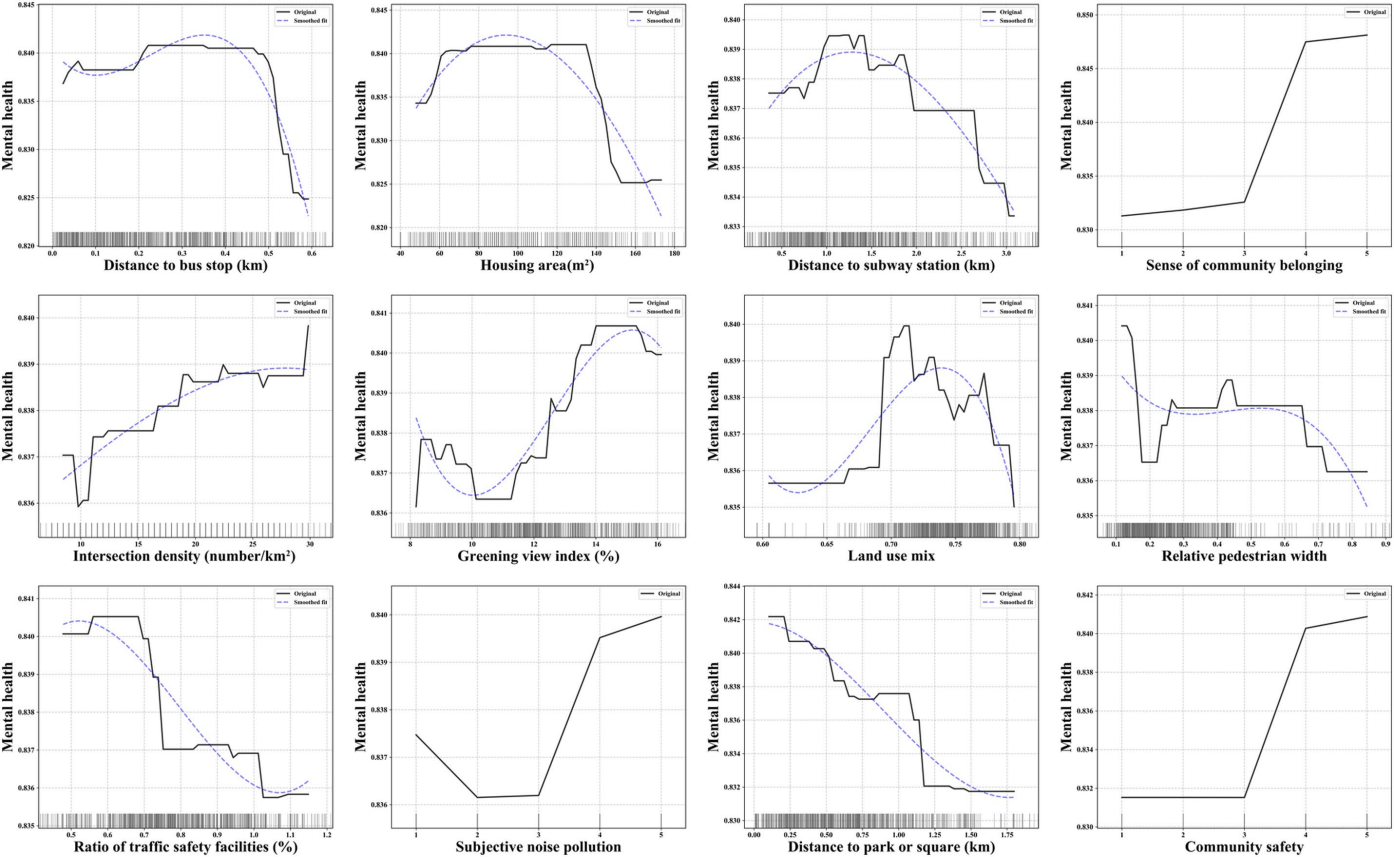
The non-linear association between key variables and adolescent mental health.

Figure 6 displays the partial dependence plots for the top 12 variables affecting adolescent mental health. In terms of the objective environment, the intersection density is positively associated with the probability of positive mental health status. Conversely, the distance to park or square, distance to bus stop, relative walking width, and ratio of traffic safety facilities are negatively associated with the probability of positive mental health status. Specifically, the probability significantly decreases when distance to bus stop exceeds approximately 0.5 km. Additionally, the distance to subway station, land use mix and greening view index exhibit differentiated relationships with the probability of positive mental health status. Specifically, the distance to subway station and land use mix lead to an “increase–decrease” trend in the probability, with a threshold at approximately 1.2 km and 0.71. The greening view index leads to a “decrease–increase” trend in the probability, with a threshold at approximately 10%.

In terms of the subjective environment, the perceived sanitary condition, subjective quality pollution, and community safety are positively associated with the probability of positive mental health status. When these three factors are rated at the highest levels, the probability of mental health is maximized.

## 5 Discussion

This study, utilizing questionnaire data and multi-source urban spatial data, employs the Random Forest model to explore the non-linear relationships between neighborhood environments and adolescent health. It assesses the relative importance of both objective and subjective environmental factors on adolescent physical and mental health and examines the impact of various variables on health status. To our knowledge, this is the first study to thoroughly explore the non-linear associations between neighborhood environments and adolescent health from both subjective and objective perspectives. The study enriches the perspectives and methods of research on neighborhood environments and adolescent health. Furthermore, it provides valuable insights for the planning of healthy communities, environmental interventions, and health promotion in specific dimensions among adolescents.

### 5.1 Contribution of the neighborhood environment to adolescent health

Compared to subjective environments, objective environments have a more significant impact on adolescent physical and mental health. This finding contrasts with existing research, which emphasizes the substantial influence of subjective environments on residents’ health and older adults health (15, 67, 68), considering them as crucial predictors of health status. Unlike adults, who possess fully developed judgment and cognitive abilities, adolescents are still undergoing developmental changes, and their mental and cognitive faculties are not yet fully mature. Therefore, their health status is more directly influenced by objective material conditions than by perceptions of social environment (e.g., neighborhood relationships, facility conditions and community image).

Additionally, we observed notable differences in the contribution of specific key variables between physical and mental health. For instance, NDVI plays a more significant role in physical health. One possible explanation is that vegetation coverage directly reflects the air quality, heat, humidity conditions (e.g., local heat island effects), and opportunities for natural contact, which are closely associated with adolescents’ respiratory and cardiovascular health (69), thereby having a more pronounced impact on physical health. In contrast, distance to bus stop and community safety are more influential for mental health. One possible explanation is that the distance to bus stop reflects the convenience of accessing social places, which plays a crucial role in the development of adolescents’ independent social capabilities and participation in activities. Similarly, during the adolescent mental development stage, fostering independent travel abilities also contributes to enhancing self-efficacy. Environmental psychology theory posits that a safe and comfortable environment can reduce anxiety and stress (70), thereby enhancing personal well-being and a sense of security. Similarly, for adolescents, the security level of the neighborhood environment (e.g., the frequency of criminal or violent incidents) indirectly influences patterns of social interaction and emotional stability, which may lead to increased anxiety and depression (63, 71, 72).

### 5.2 The impact of objective environment on adolescent health

#### 5.2.1 Non-linear relationship between the objective environment and adolescent health

The presence of non-linear relationships and threshold effects between key objective environmental variables and adolescent physical and mental health is notable. For instance, the land use mix leads to an “increase–decrease” trend in the probability of adolescent health. An appropriate level of land use mix reflects the diversity of public facilities, potentially reducing the need for long commutes and promoting active travel modes (e.g., walking and cycling) (73, 74), thereby enhancing physical health. Furthermore, social interactions within diverse public spaces can facilitate expansion of social networks, and the development of self-confidence. However, an excessive degree of land use mix often leads to high-frequency, prolonged population activities within the community, creating a bustling neighborhood atmosphere that may negatively impact both sleep quality and academic performance.

The distance to park or square is negatively associated with physical and mental health, while the NDVI is positively associated with physical health. This observation aligns with the biophilia hypothesis, which describes humans’ intrinsic connection to nature and an innate tendency for outdoor exploration (75). Adolescents foster positive health outcomes through participation in physical activities, nature exposure, and social interactions within natural or open spaces (6, 28, 29). Furthermore, high-quality outdoor environments (e.g., good air circulation and low noise levels) benefit respiratory and physical health (21, 22). The relatively smooth curve observed for NDVI suggests that the health-promoting effects of nature are more evident in well-maintained, landscaped natural and open spaces.

The probability significantly decreases when distance to bus stop exceeds approximately 0.5 km. The 0.5 km radius around a bus stop is generally sufficient to meet residents’ travel needs and is a fundamental feature of a 5-min pedestrian-scale neighborhood (76). Therefore, the distance may not directly affect adolescents’ mood through promoting physical activity. However, when bus stops are located farther than 0.5 km, transportation inconvenience restricts adolescents’ opportunities and willingness to engage in outdoor social activities. This reduced accessibility may foster feelings of anxiety and social isolation, ultimately exerting a negative impact on mental health.

The relative pedestrian width is negatively associated with physical and mental health. In urban road design, sidewalk width is often closely related to the road classification. As a result, wider sidewalks are typically found along primary and secondary arterial roads, which are less likely to be chosen for active commuting by residents (77). For instance, as demonstrated in this study, relative pedestrian width exceeding 0.4 no longer shows a significant impact on physical health. While wider pedestrian spaces provide opportunities for social interaction (e.g., certain sidewalk widths may promote mental health), the higher traffic flow on major roads often presents perceived safety risks and psychological stress for adolescents when walking or crossing these roads.

The intersection density is positively associated with mental health. Communities with higher intersection density generally feature a more diverse distribution of facilities and more active interpersonal interaction patterns (e.g., with more flexible land use layouts). Consequently, in such high-access environments, adolescents can more easily and freely access social activity locations. The social interaction opportunities provided help alleviate stress, improve life satisfaction, and foster a sense of community belonging.

The sky view index leads to a “decrease–increase” trend in the probability of physical health. Low levels of sky openness create a sense of spatial enclosure, resulting in visual interference with traffic-related travel activities on the streets, thereby reducing adolescents’ willingness to engage in active travel. However, similar to existing studies, as the spatial view gradually opens up, active physical activities such as walking and cycling are more likely to occur on the streets (78, 79), thereby promoting individuals’ physical fitness, cardiovascular health, and overall well-being.

#### 5.2.2 Differences in the impact of objective environment on adolescent physical and mental health

It is noteworthy that some variables exhibit significant differences in their impact on physical versus mental health. Firstly, compared to physical health, the distance to subway station exhibits a clear trend of initially increasing and then decreasing. At an optimal proximity to the subway station (approximately 1.2 km), adolescents can conveniently access entertainment, sports, and recreational spaces through the “subway +” travel mode, which in turn enhances social connections, reduces feelings of loneliness, and improves life satisfaction. However, the distribution of subway stations reflects urban location differences and functional layout needs, with demand primarily concentrated on long-distance commuting rather than daily short trips, thus failing to significantly promote travel modes that support physical activity. Furthermore, as the distance to the subway station increases, the probabilities of both physical and mental health decline. On one hand, this result suggests that greater distances from transportation (including bus stops and subway stations) limit opportunities for social participation, leading to “social isolation.” On the other hand, increased commuting time reduces opportunities for active commuting to school and outdoor physical activities, resulting in an overall decrease in adolescents’ physical activity, which negatively impacts physical health. This result confirms that, beyond the positive effects of transportation stations within a fixed proximity (7, 13), excessive distance may even have negative effects.

Secondly, the ratio of traffic safety facilities is positively associated with physical health, while negatively associated with mental health. Well-established traffic safety features (e.g., traffic lights, barriers, curbs) regulate pedestrian spaces and behaviors on streets (80), which reduces the risk of injury or traffic accidents for adolescents and encourages them to actively choose walking, cycling, and other modes of participation in street activities. However, an excessive number of traffic safety facilities can introduce environmental warnings, visual complexity, and a sense of safety dependence. On one hand, this may cause confusion among adolescents about the external environment and a sense of helplessness regarding potential dangers. On the other hand, the overly “formal” atmosphere created by these facilities hinders adolescents from developing an exploratory desire based on the complexity and diversity of the environment.

Thirdly, compared to physical health, the greening view index significantly induces a “decrease–increase” trend in mental health within certain intervals. A possible explanation is that in areas with low levels of street greening, adolescents are more likely to feel mentally oppressed or uncomfortable (e.g., high levels of spatial construction intensity) and unsafe (e.g., vast open spaces) due to the lack of natural exposure, which can lead to negative emotions. As the level of greenery increases (particularly after exceeding 10%), both the physical and mental health of adolescents gradually improve. This further demonstrates that, in addition to the proximity and coverage of green spaces within neighborhoods, well-visible green spaces on streets can also promote health through environmental contact and activity (expressed through active commuting behaviors) (62, 81). However, excessively high levels of green visibility imply excessive obstruction of street space and a lack of construction activity, which leads to fewer adolescents engaging in physical activities or social interactions in these areas.

### 5.3 The impact of subjective environment on adolescent health

We found that the subjective environment typically has a beneficial impact on promoting adolescent health. For instance, among the key variables related to community management, such as community safety is positive associated with adolescent mental health. Poor maintenance of facilities, littering, and other signs of environmental disorder can lead to increased concerns about public safety and crime (71, 72). In contrast, a well-maintained and safe community environment enhances adolescents’ sense of security and comfort during outdoor activities, which can alleviate potential sleep difficulties and reduce life stress (6).

Among the key variables related to community image, a quieter noise environment is beneficial to physical and mental health. Previous studies have indicated that urban traffic noise leads to sleep disturbances (82). Similarly, exposure to high levels of noise (including traffic and activity noise) can induce stress responses in adolescents, potentially affecting academic performance and sleep quality. Air quality can directly affects respiratory and cardiovascular health (69). Exposure to low levels of air pollution (e.g., NO_2_, PM_2.5_), is more likely to lead to physiological issues such as insomnia and cardiovascular diseases during adolescence (6, 23).

Among the key variables related to community capital, sense of community belonging positively influences both adolescent physical and mental health. Existing research indicates that social cohesion among neighbors helps alleviate psychological stress and enhances social connections in adults (83, 84). Similarly, a supportive and emotionally connected neighborhood environment promotes positive health behaviors and psychological well-being in adolescents (40, 85).

### 5.4 Limitations

This study has two limitations due to the study area and sample constraints. First, due to the sample size of the survey data, it is challenging to control for geographic environmental differences that may arise from residential self-selection (16). For instance, parents of students living in commercial properties or rental accommodations might choose residences with better scenic environments and amenities based on their economic status and social position. Second, although we explored the optimal impact range of the built environment on adolescent physical and mental health, the threshold effects of the built environment may differ under varying regional conditions. Therefore, future research should aim to enhance the generalizability of the findings by conducting comparative studies across different cities and increasing sample size. Nonetheless, considering the challenges of data acquisition, the non-linear and threshold effects of neighborhood environments highlighted in this study offer valuable insights for adolescent-friendly community planning, construction, and management, particularly for large cities along the southwestern border of China.

## 6 Conclusion

This study, leveraging survey data, multi-source urban spatial data, and machine learning techniques, examines the relative importance of subjective and objective environmental factors on adolescent physical and mental health, and analyzes the non-linear relationships between various environmental variables and health status. The findings reveal that: (1) Objective environments exert a greater influence on adolescent physical and mental health compared to subjective environments. (2) Within objective environments, the relationship between different variables and adolescent health is typically non-linear, displaying threshold effects. Notably, the distance to subway station, ratio of traffic safety facilities, and greening view index have differing impacts on physical and mental health. (3) Within subjective environments, key factors related to community management, community image, and community capital generally positively influence adolescent health.

Based on the above conclusions, this study explores three aspects of interventions in the neighborhood environment (including both subjective and objective elements), and aims to provide policy recommendations for policymakers, planners, and community managers in planning and managing of healthy communities. Firstly, in urban planning and the layout of community facilities, policymakers and planners should consider flexible facility models, with an emphasis on the rational arrangement of landscape spaces, open spaces, and transportation facilities. For instance, considering the health-promoting effects of green landscapes and open spaces, the construction of community parks, gardens, and playgrounds should be prioritized (86). A distance exceeding 450–500 meters from bus stations has been associated with reduced adolescent health levels. Therefore, their layout should ensure a service radius of 500 meters within the 5-min pedestrian-scale neighborhood. Secondly, in the renovation of community street microenvironments, planners should consider the nonlinear impacts and potential threshold effects of three-dimensional spatial boundaries on adolescent health. For instance, excessively high greenery coverage (over 14%) no longer significantly impacts physical and mental health, while low sky openness (below 52%) is detrimental to health. Therefore, urban planners should adopt refined spatial strategies in street renovation and optimization for these specific environmental elements. Thirdly, community management efforts should focus on enhancing a positive community atmosphere. Given the general health-promoting effects of the neighborhood’s perceived environment on adolescent health, policymakers and community managers should focus not only on improving the provision of “hardware” such as landscape environments, sanitation conditions, and security facilities but also on fostering a supportive “software” atmosphere, including social cohesion, networks, and support.

## Data Availability

The raw data supporting the conclusions of this article will be made available by the authors, without undue reservation.

## Footnotes

^1^ https://www.openstreetmap.org/

^2^ https://lbs.amap.com/

^3^ https://lbsyun.baidu.com/

^4^ http://www.nesdc.org.cn/
